# Changed Genome Heterochromatinization Upon Prolonged Activation of the Raf/ERK Signaling Pathway

**DOI:** 10.1371/journal.pone.0013322

**Published:** 2010-10-12

**Authors:** Catherine Martin, Songbi Chen, Daniela Heilos, Guido Sauer, Jessica Hunt, Alexander George Shaw, Paul Francis George Sims, Dean Andrew Jackson, Josip Lovrić

**Affiliations:** 1 Faculty of Life Sciences, Manchester Interdisciplinary Biocentre, The University of Manchester, Manchester, United Kingdom; 2 Tropical Crops Genetic Resources Institute, Chinese Academy of Tropical Agricultural Sciences, Hainan, China; 3 Max Planck Institute for Developmental Biology, Tübingen, Germany; Texas A&M University, United States of America

## Abstract

The Raf/ERK (Extracellular Signal Regulated Kinase) signal transduction pathway controls numerous cellular processes, including growth, differentiation, cellular transformation and senescence. ERK activation is thought to involve complex spatial and temporal regulation, to achieve a high degree of specificity, though precisely how this is achieved remains to be confirmed. We report here that prolonged activation of a conditional form of c-Raf-1 (BXB-ER) leads to profound changes in the level and distribution of a heterochromatic histone mark. In mouse fibroblasts, the heterochromatic trimethylation of lysine 9 in histone H3 (H3K9Me3) is normally confined to pericentromeric regions. However, following ERK activation a genome-wide redistribution of H3K9Me3 correlates with loss of the histone modification from chromocentres and the appearance of numerous punctuate sites throughout the interphase nucleus. These epigenetic changes during interphase correlate with altered chromosome structure during mitosis, where robust H3K9Me3 signals appear within telomeric heterochromatin. This pattern of heterochromatinization is distinct from previously described oncogene induced senescence associated heterochromatin foci (SAHF), which are excluded from telomeres. The H3K9Me3 histone mark is known to bind the major heterochromatin protein HP1 and we show that the alterations in the distribution of this histone epistate correlate with redistribution of HP1β throughout the nucleus. Interestingly while ERK activation is fully reversible, the observed chromatin changes induced by epigenetic modifications are not reversible once established. We describe for the first time a link from prolonged ERK activation to stable changes in genome organization through redistribution of heterochromatic domains involving the telomeres. These epigenetic changes provide a possible mechanism through which prolonged activation of Raf/ERK can lead to growth arrest or the induction of differentiation, senescence and cancer.

## Introduction

Cells respond to extra-cellular cues by activating signal transduction networks like the Raf/ERK pathway, which is downstream of receptor tyrosine kinases. As a consequence of the activation of growth factor receptors, Ras is present in its active form on the inner plasma membrane. Raf kinases bind to activated Ras as part of their complex activation mechanism. Active Raf in turn phosphorylates and activates MEK 1/2 (Mitogen activated Protein Kinase-/Extracellular Signal Regulated Kinase-Kinase) which in turn phosphorylate and activate ERK1/2 (for a recent review see [1]). Other elements of regulation of the Ras/Raf/MEK/ERK pathway include homo and heterodimerization of a variety of proteins as well as both activating and inhibitory phosphorylations. The ERK pathway also shows a variety of feedback inhibition loops, autocrine regulation and an extensive crosstalk to other signaling modules like PI3(Phosphoinositide 3) kinases or PKCs (Protein Kinase C). ERKs are the workhorses of this signaling cascade and have more than 160 cellular substrates [2].

Whilst the known ERK substrates are spread over several cellular compartments and localizations, special attention has historically be paid to nuclear substrates. Activated ERKs can accumulate in the nucleus [3] where modification of transcription factors can drive gene expression [2], [4]. The cumulative phosphorylation of ERK substrates regulates numerous cellular events including cell transformation and tumor development [3], [5]–[7]. At the moment, it is not well understood how the activation of such a ubiquitous pathway, with a multitude of substrates, can have the very specific cellular outcomes observed in a variety of cellular model systems. Signal duration is one regulatory element defining biological function [4], although how prolonged ERK activation induces irreversible effects like senescence and differentiation (as opposed to growth following short term activation) is not known and a matter of controversy [8]–[11]. Interestingly, in PC12 cells prolonged ERK activation, as induced by NGF (Neuronal Growth Factor), induces differentiation, while short term ERK activation, induced by EGF (Epidermal Growth Factor), causes growth. Proteins preferentially interacting with ERK after NGF stimulation are involved in a variety of cellular processes like signaling, apoptosis regulation, protein transport and metabolism; just a minor portion are transcriptional regulators [12]. This indicates that processes outside transcriptional regulation might be important factors conferring signaling specificity in the ERK pathway. It was also shown that, apart from the induction of immediate early genes via the phosphorylation of transcription factors, ERK activation can lead to long-term reprogramming of gene expression through alterations in chromatin organization and DNA methylation [4], [13]–[15].

A preliminary proteomics study in our laboratory (unpublished) identified several proteins involved in the regulation of chromatin and nuclear structures as targets of Raf signaling. Amongst those proteins were RNF2 (Ring Finger 2), nuclear lamins and HP1 (Heterochromatin Protein 1) proteins. These preliminary studies showed that prolonged activation of Raf/ERK influenced proteins involved with the epigenetic histone code. The interaction between DNA, chromatin and chromatin associated factors are very complex and regulate functions in information maintenance and transcriptional regulation.

Epigenetic chromatin marks (mainly modifications of histone tails) as well as the methylation state of the DNA regulate DNA interactions with chromatin associated factors, defining chromatin patterns that correlate with transcriptional status [16]. Different epigenetic marks define patterns of chromatin folding that are permissive or repressive for gene expression. The corresponding chromatin states, euchromatin and heterochromatin, are faithfully transmitted to daughter cells during mitosis, in order to maintain epigenetic programming [17]. As an essential component of global chromatin organization, pericentric heterochromatin plays a fundamental role in both, nuclear organization and gene silencing. It is made up of tandem repeats of a 234 bp A-T rich motif, called the major satellite, constituting a domain which directly flanks the inner centromere. Interestingly during interphase, pericentric satellites from different chromosomes gather and form large domains called chromocenters, which are particularly prominent in murine cells. These centers display specific and robust epigenetic marks, which are maintained through many cell cycles [18]. In this chromatin compartment, the compact chromatin, which is ‘glued’ by many silencing factors, creates an environment that is highly repressive for transcription. This is evident during cell differentiation, when inactive genes are often seen to lie in the vicinity of heterochromatic domains [19]–[22]. Hence, nuclear remodeling and chromatin dynamics are key features of cell differentiation [23] and the organization of heterochromatic domains contributes to this process [24].

Silencing at chromocenters can result directly from their unique epigenetic patterns of post-translational histone modifications. Indeed, histone methyltransferases homologous to Su(var)3–9 specifically methylate lysine 9 of histone H3 at pericentric satellites (H3K9Me3, [25]). The α and β isoforms of HP1 also bind to the modified histone tails and altogether these marks contribute to maintenance and propagation of heterochromatin at chromocenters [26], [27]. HP1 is a 25 kDa protein, which is ubiquitous in eukaryotes [28], [29]. Three domains are responsible for the complex functions of HP1 [26]: *i)* a “chromo” domain (for ‘chromatin-organization modifier’) which recognizes the H3K9Me3 [30], [31]: *ii)* a “hinge” domain that binds RNA and DNA [32], [33] and *iii)* a “chromoshadow” domain, which facilitates HP1 dimerization and thereby heterochromatin propagation. In D. melanogaster HP1 is known to be a key determinant of gene silencing in the vicinity of heterochromatin blocks [34] and the protein is thought to play a similar role in mammalian cells.

Following our preliminary observations, the aim of this study was to evaluate if Raf/ERK activation can alter global nuclear organization, particularly the structure of major chromatin compartments in mouse fibroblasts, using a conditional form of Raf, BXB-ER [35]. BXB-ER is a fusion protein of the human estrogen receptor hormone binding region and the carboxy-terminal half (containing the kinase domain) of human c-Raf-1, which is only active in the presence of estrogen. Taking advantage of the targeted activation of the BXB-ER activity, we observed that activation of Raf resulted in profound changes in the distribution of heterochromatin, which was seen to spread to sites throughout the genome and notably at telomeres. The re-organization of the heterochromatin specific epigenetic marker H3K9Me3 was mimicked by changes in the distribution of the heterochromatin binding protein HP1. Moreover, the nuclear changes seen during Raf/ERK activation were shown to correlate with alterations in cell cycle progression and the onset of p21 induced cell cycle arrest. This implies that changes in the organization of heterochromatin contribute to the cell cycle arrest that is induced by prolonged activation of the Raf/ERK pathway.

## Results

**Figure 1:**
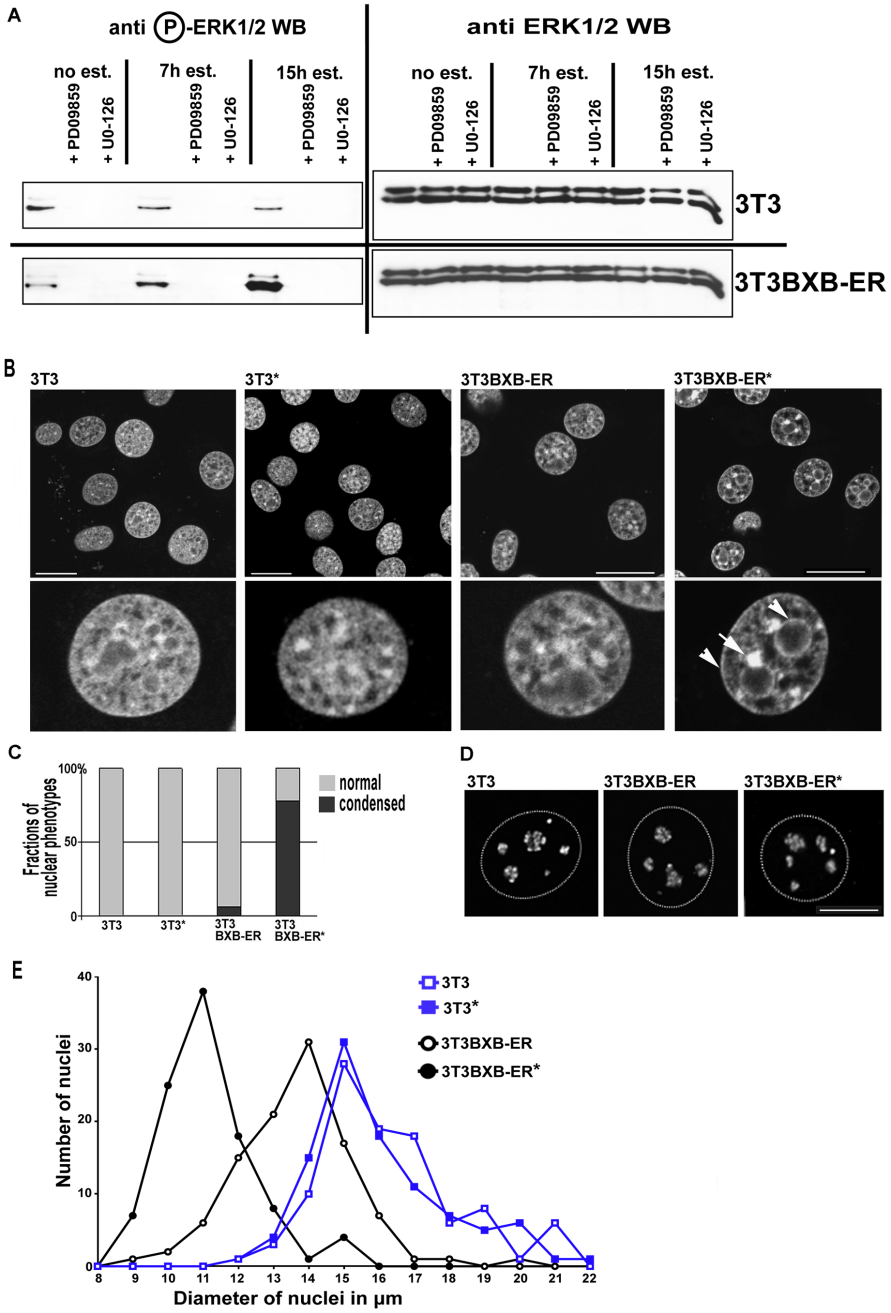
BXB-ER activation induces chromatin rearrangement and reduced nuclear diameter. (A) Western blot of 3T3 and 3T3BXB-ER cells for phosphorylated ERK1/2 (left panel) and for total ERK1/2 (right panel). Cells were treated with estrogen for 7 hours or 15 hours as indicated. PD09859 and UO-126 were added either 30 sec before estrogen was added (7 h est. and 15 h est.) or 15 hours before the cells were harvested (no est.). 20 µg of protein from the various whole cell lysates were loaded into each lane. (B) Optical sections of control 3T3 and 3T3BXB-ER cells stained with Sytox green to show DNA distribution. Enhanced condensed chromatin clumps (arrow) and rims surrounding nucleoli and nuclear periphery (arrowheads) after BXB-ER activation by addition of estrogen for 15 hours (*) are indicated. Scale bars: 20 µm. (C) Proportions of normal (grey) and condensed (black) chromatin phenotypes after analysis of ∼100 cells per condition. “normal” nuclei are defined by the absence of a nuclear rim, the absence of rims surrounding the nucleolei and the absence of condensed nuclear clumps. “condensed” nuclei were defined by the presence of at least one of these features. Nearly all condensed nuclei displayed all three features at the same time. (D) 3D reconstructions of cells immunoprocessed for fibrillarin [38] show no changes in nucleolar structure. The dotted lines indicate the outline of nuclei. Scale bars: 10 µm. (E) Size distribution of nuclei in untreated and estrogen treated 3T3 and 3T3BXB-ER cells. The mean diameters of 100 nuclei for each condition were determined and sorted in 1 µm wide bins.

Control experiments were performed to ensure that BXB-ER does indeed activate MEK which then results in activation of ERK1/2 in our system just as shown before [35]. We also wanted to control that the estrogen used for activating the kinase activity of BXB-ER has no effect on ERK activity in 3T3 cells. For this, 3T3 control cells and 3T3BXB-ER cells were treated for 7 and 15 hours with estrogen and ERK1/2, activated by phosphorylation on residues Thr 202 and Tyr 204, was detected by Western blot (Fig. 1A). There was a small amount of activated ERK1/2 in untreated 3T3 cells and this was not influenced by estrogen treatment. When cells were treated with 2 different inhibitors of the Raf and BXB-ER mediated MEK activation, PD09859 and UO-126 [36], [37], the endogenous level of ERK1/2 activity was suppressed completely. Similar to 3T3 cells, 3T3BXB-ER cells also showed a small amount of phosphorylated ERK1/2 in the absence of estrogen. However, in contrast to the situation in 3T3 cells, 3T3BXB-ER cells expressing BXB-ER showed enhanced ERK1/2 activity after 7 hours of estrogen treatment and ERK1/2 activity was further increased after 15 hours of estrogen treatment. Analysis of total ERK1/2 amounts by Western blot showed that the regulation of ERK1/2 activity is mainly on the level of protein phosphorylation, as no global changes in the abundance of ERK1/2 were seen under any of the treatment condition (Fig. 1A).

Nuclear structure and distribution of heterochromatin in 3T3BXB-ER cells and in control 3T3 cells were monitored by DNA staining (Fig. 1B). 3T3 cells, 3T3 cells treated with estrogen and untreated 3T3BXB-ER cells displayed classical chromatin organization with dispersed euchromatin punctuated by a small number of discrete brightly-stained clumps of heterochromatic chromocenters and some small dense chromatin clumps along the nuclear periphery and around nucleoli (Fig. 1B). Cells with activated BXB-ER (and thus activated MEK/ERK Fig. 1A and [35]) displayed a heterogeneous texture with more dense chromatin aggregates (Fig. 1B, arrows) and discrete chromatin foci along the nucleolar periphery and nuclear lamina (Fig. 1B, arrowheads). Quantification of several hundred nuclei showed that this phenotype is predominant in activated BXB-ER nuclei and essentially absent in the nuclei of control cells including 3T3 cells treated with estrogen (Fig. 1C). 3T3BXB-ER cells show minor changes in the structure of heterochromatin when compared with 3T3 control cells, consistent with a slight “leakiness” of the conditional Raf, which was also observed in previous studies [35].

The chromatin changes we observed following BXBER activation correlated with an overall decrease in nuclear diameter in 3T3BXB-ER cells after 15 hours of BXB-ER activation (Fig. 1D/E). Measuring the nuclear diameters from 100 cells for each sample, 3T3 nuclei showed average diameters of 16.66+/−1.86 µm for cells treated with ethanol (carrier for estrogen) alone and 16.57+/−2.08 µm after 15 hours of estrogen treatment. The nuclei of BXB-ER cells on the other hand were measured at 14.09+/−1.52 µm and at 11.76+/−1.55 µm for ethanol/estrogen treated cells respectively. The differences between the values from the 3T3BXB-ER cells as well as the differences between 3T3 and 3T3BXB-ER cells were highly significant (p≤0.001), resulting in a decreased nuclear volume by ∼40% in estrogen treated BXB-ER cells. Measurements of the size distribution of the nuclei from 3T3 and 3T3BXB-ER cells after estrogen treatment (Fig. 1E) showed that changes in cell cycle distribution can not be a major contributing factor for the reduced nuclear diameter in 3T3BXB-ER cells after estrogen treatment, as the peak value of nuclear diameters of estrogen treated BXB-ER cells is completely outside the range of nuclear size distribution in 3T3 cells. We conclude that activated BXB-ER (and thus prolonged ERK activation) leads to a significant decrease in nuclear size in 3T3BXB-ER cells, which is not directly linked to their cell cycle distribution. Untreated 3T3BXB-ER cells also have a slight decrease in nuclear size compared to 3T3 cells. This partial phenotype is consistent with some leakiness of the BXB-ER construct (see also [35]).

As changes in nuclear volume often correlate with alteration in functional status, we monitored the distribution of fibrillarin in nucleoli. Fibrillarin is a marker for active transcription centers in nucleoli and stains coiled bodies, which show as bright single spots, providing a marker for global nucleolar organization and transcriptional activity [38]. BXB-ER activation had no effect on the number or structure of nucleoli or their active centers (Fig. 1D). Thus, the reduced nuclear volume in cells with activated BXB-ER correlated with global condensation of heterochromatin and genome compaction, even though the transcriptional status of nucleoli is unaltered. Interestingly, a previous study [35] showed that BXB-ER activation also induces bundling of the cytoskeletal microtubules and an elongated cell shape. These microtubular changes correlate with changes of the cell shape; the predominantly flat 3T3 BXB-ER cells become more elongated and develop a spindle-shaped morphology after estrogen treatment, while 3T3 show no changes after estrogen treatment [35]. We have no indication that these morphological changes in 3T3BXB-ER result in changes of cellular volume, as we never observed a change in the volumes of cellular pellets after estrogen treatment (data not shown). Also the fresh weight of cellular pellets does not show any statistically significant differences after estrogen treatment, with average values of 31.1 +/− 5.7 pg/cell for control 3T3 cells and 46.6 +/− 19.2 pg/cell for estrogen treated 3T3 cells. The values for control and estrogen treated 3T3BXB-ER cells were 38.5 +/− 22 pg/cell and 28.0 +/− 9.5 pg/cell respectively (see Material and Methods). In summary whilst we can not exclude minor changes in cellular volumes during our experiments, the dramatic changes in nuclear volume after BXB-ER activation are not accompanied by obvious changes in the overall volumes of the cells.

**Figure 2:**
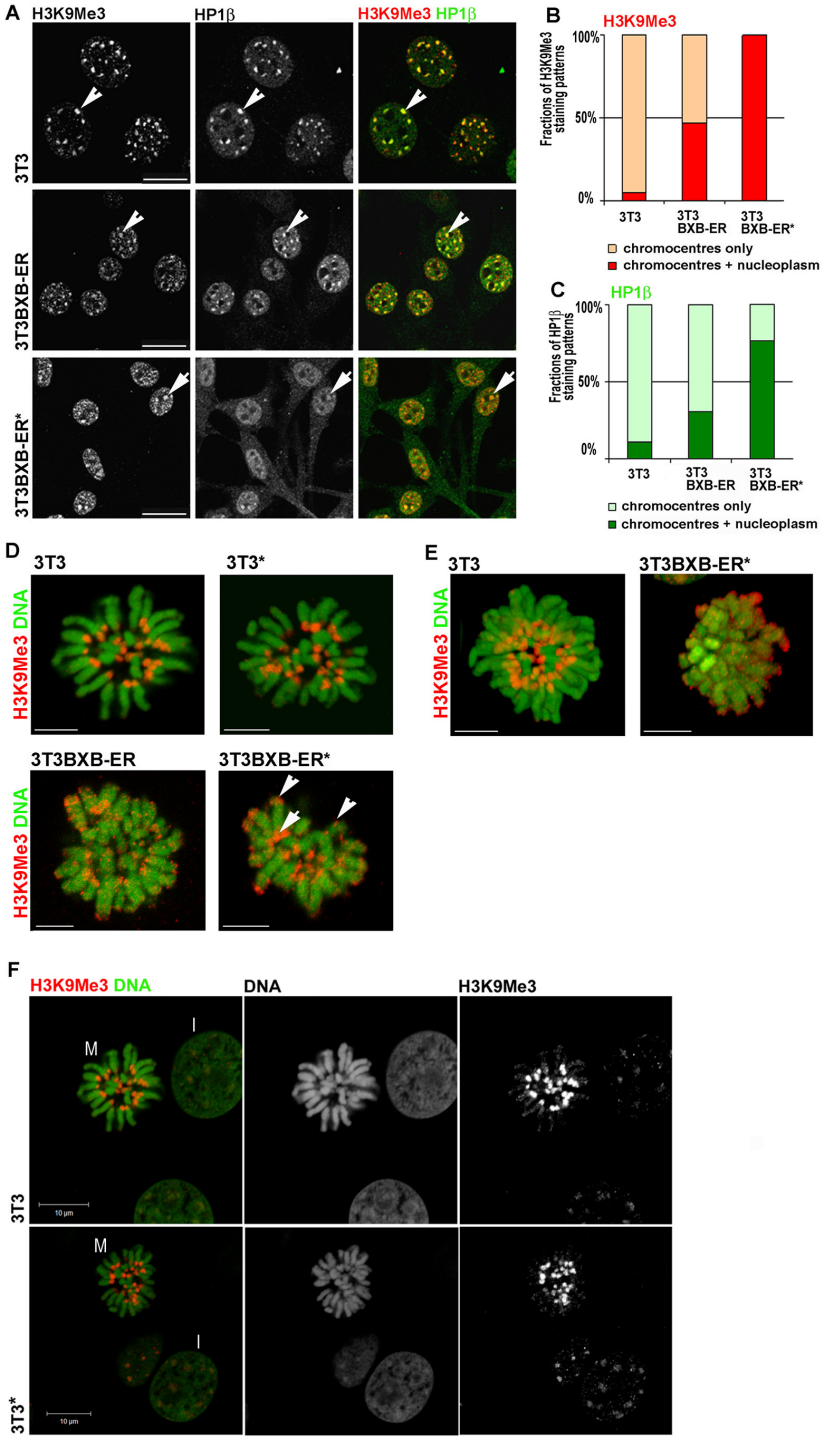
BXB-ER activation induces changes in H3K9Me3/HP1 distribution between chromocentres and nucleoplasm and spreads H3K9Me3 to telomeres. (A) Optical sections of cells that were immunoprocessed for both H3K9Me3 (red in double stains) and HP1β (green in double stains) as indicated above the panels [77]. The staining pattern reveal the spreading of H3K9Me3 and HP1β to numerous nucleoplasmic sites upon BXB-ER activation. Normal chromocentres with co-localized H3K9Me3/HP1β appear yellow (arrowheads) and chromocentres with reduced HP1® appear red (arrows). Individual immunoflorescences are shown in grey. Scale bars: 20 µm. (B/C) Proportions of cells with immunofluorescence restricted to chromocentres versus cells with additional nucleoplasmic signals were scored (223 and 129 cells for HP1β and H3K9Me3, respectively). (D/E) Metaphase spreads from indicated cells, after growing without or with estrogen (*) for 15 hours, were immunoprocessed for H3K9Me3 (red) and DNA was counterstained with Sytox (green). Optical slices (D) and corresponding 3D reconstructions (E [38]) show normal pericentric H3K9Me3 staining (arrow) and new distinct, small sites of H3K9Me3 on chromosome arms and telomeres (arrowheads) after activation. Scale bars: 5 µm. (F) Control experiment to show that estrogen treatment has no influence on the pattern of H3K9Me3 distribution in interphase or mitotic 3T3 cells. Untreated 3T3 cells or 3T3 cells treated for 15 hours with estrogen (*) were immunoprocessed for H3K9Me3 (red) and DNA was counterstained with Sytox (green). Optical slices of fields showing metaphase spreads (M) and interphase cells (I) are shown. Scale bars: 10 µm.

To characterize potential chromatin rearrangements following BXB-ER activation we analyzed the distribution of epigenetic markers associated with pericentric heterochromatin using antibodies to the heterochromatic histone modification H3K9Me3. In the acrocentric mouse chromosomes, pericentric heterochromatin is also sub-telomeric. The pericentric heterochromatin aggregates into ∼10 chromocenters per nucleus, which are known to be very prominent in murine cells [27], [39]. Immunostaining of 3T3 cells and untreated 3T3BXB-ER showed that H3K9Me3 was restricted to the heterochromatin within chromocentres (Fig. 2A). In addition, chromocentres were enriched for the H3K9Me3 associated heterochromatin protein 1 β (HP1β; Fig. 2A). However, activation of BXB-ER induced a diminished H3K9Me3 staining at chromocentres and a dramatic increase in dispersed H3K9Me3 staining throughout the nucleus (Fig. 2A), while no changes in H3K9Me3 staining patterns could be observed in estrogen treated 3T3 cells (Fig. 2F). Heterochromatin associated HP1β binds strongly and specifically to H3K9Me3 [26]. We analyzed the distribution of HP1β in control cells and cells with activated BXB-ER (Fig. 2A). The HP1β staining pattern was almost identical to the staining pattern of H3K9Me3. However, the phenotype of the loss of structure for the staining pattern of heterochromatin associated HP1 was even more pronounced than that of H3K9Me3 in cells with activated BXB-ER, leading to some chromocentres showing a slightly greater loss of HP1β compared to H3K9Me3 (Fig. 2A, arrow). Changes were quantified by scoring the distribution of patterns of H3K9Me3 staining (Fig. 2B) and HP1β staining (Fig. 2C) at chromocentres and in the nucleoplasm of several hundred individual cells (see Material and Methods). 3T3 cells showed no differences in staining patterns after estrogen treatment (Fig. 2F) and are included in the quantification as a reference.

To assess the genome-wide location of the H3K9Me3 distribution at higher resolution, we next analyzed chromatin distribution in mitotic cells (Figs. 2D/E). In control 3T3 cells H3K9Me3 exclusively decorated the pericentromeric regions of chromosomes (Fig. 2D). In untreated 3T3BXB-ER cells we observed some minor additional sites of H3K9Me3 decoration. However, following activation of BXB-ER an obvious increase in staining was seen on specific locations throughout the chromosome arms (Fig. 2D). Notably, the altered staining pattern decorated sub-chromosomal bands on many chromosomes (Fig. 2D) and strongly stained telomeres, which lie distal to the pericentromeric heterochromatin (e.g. Fig. 2D, arrowhead). 3D reconstructions of the chromosomal structures showed the overall spatial architecture of the chromosomes (Fig. 2E). The 3D structures clearly showed the pericentromeric heterochromatic patterns in normal 3T3 cells and that the normal chromosomal architecture in metaphase cells was lost in cells with activated BXB-ER (Fig. 2E). Activated BXB-ER also resulted in swollen or fluffy chromosome structure, so that discrete daughter chromatids were no longer evident. The reorganization of H3K9Me3 correlated with a deterioration of chromosome organization and orientation on the metaphase plate (Figs. 2D/E). In contrast to the changes following the activation of BXB-ER with estrogen, no changes at all could be detected in the H3K9Me3 staining pattern in mitotic 3T3 cells after the treatment with estrogen (Fig. 2F).

**Figure 3:**
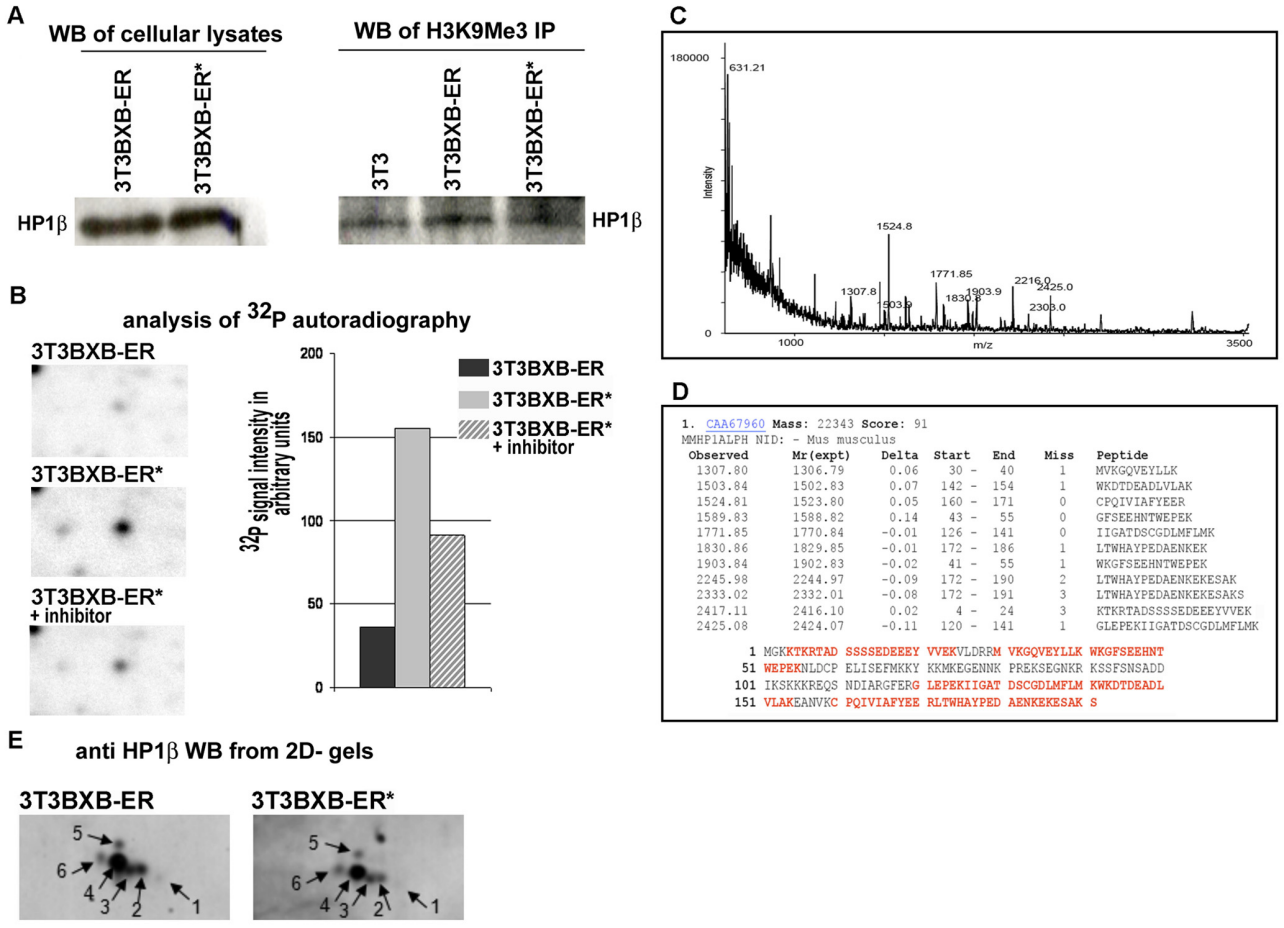
HP1/H3K9Me3 interaction is unaffected by HP1 phosphorylation following BXB-ER activation. (A) Western blot for HP1β of untreated and 15 hours estrogen treated (*) 3T3BXB-ER cells. Lysates in the “H3K9Me3 IP” (right hand) panel were immunoprecipitated using an antibody specific for H3K9Me3 and Western blotted for HP1β. (B) Autoradiograph of ultra high resolution 2D SDS PAGE gels (pH 4.5–5.5, region shown around pH 4.9, MWr ∼22 kD) of lysates from cells labeled metabolically with ^32^P [35]. Cells were either untreated, activated with estrogen for 20 min (*) or activated and treated with PD98059 at the same time (* + inhibitor). The spot in the middle of the panels showed reproducible changes in phosphorylation intensity in 3 experiments, the one shown is a representative. Bars show relative intensities of the phosphorylation intensity of this spot, relative to all spots on the gel. This spot was later identified as HP1α (see C/D). (C/D) Spots shown in (B) were identified by Peptide Mass Fingerprinting. For this the spots were cut out from the gel, digested with trypsin and processed for identification by MALDI ToF-MS [35]. Panel C shows the raw data from the MALDI ToF MS. Peaks were manually picked and submitted to a MASCOT (Matrix Science, UK) search of the non-redundant MSDB database. (D) Murine HP1α was identified as the best match to the sequences in the database with a MASCOT score of 91 (scores >70 denoted statistical significance at the 0.05% level). Mass accuracy was set to 100 ppm and three missed tryptic cleavages were allowed, as HP1α contains a higher than normal amount of Arg (R) and Lys (K). Panel D also shows the 59% sequence coverage of the matching peptides in red. Sequence coverage went down to 46% with more typical setting of one missed tryptic cleavage allowed (not shown). (E) Western blot for HP1β from high resolution 2D SDS PAGE gels (pH 3–5.6, region shown around pH 3.8, apparent MW ∼22 kDa). The spots shown are the only signals generated from a much larger Western blot. Spot 1 is the least acidic spot recognized by the HP1β antibody, with the smallest apparent MW. The unnumbered spot is an artifacts, unique to this experiment. 5 experiments with similar results were performed.

The spreading of heterochromatic histone marks on chromosomes as evident from Figs. 2D/E is clearly consistent with the large number of nucleoplasmic H3K9Me3 sites that are seen in interphase cells following BXB-ER activation (Figs. 2A/B). To investigate the impact of alterations in the H3K9Me3 distribution we analyzed the HP1 proteins HP1α and HP1β, which are known to interact with H3K9Me3 in chromatin and can have slightly different localizations within euchromatin and heterochromatin [26]. Western blotting revealed that expression of HP1β was essentially unchanged following BXB-ER activation (Fig. 3A). To establish if the association of HP1β with H3K9Me3 was affected by BXB-ER activation we probed immunoprecipitates of H3K9Me3 for the presence of associated HP1β by Western blotting. These experiments showed that the association of HP1β to H3K9Me3 was unaltered in 3T3BXB-ER cells, regardless of BXB-ER activation and was essentially the same as in 3T3 cells (Fig. 3A).

Phosphorylation of HP1 proteins dictates their role in gene silencing [40], [41] as well as their nuclear localization during the cell cycle [42] and can influence their association with H3K9Me3 [43]. HP1β interacts in heterochromatin with other family members, like HP1α, to regulate the structure and function of heterochromatin [26]. We thus assessed if changes in phosphorylation of HP1α or HP1β might account for the altered localization following BXB-ER activation we observed for HP1β. Proteomic analysis of ^32^P labeled phosphorylated proteins from 2-D PAGE gels revealed a protein whose phosphorylation was increased within 20 min of activation of BXB-ER (Fig. 3B). This protein was identified as Hp1α using Peptide Mass Fingerprinting (Figs. 3C/D). In contrast, Western blots of 2-D PAGE gels showed no major changes in post-translational HP1β isoform patterns (Fig. 3E; 6 isoforms marked). Based on its isoelectric point and its relation to the other HP1β isoforms detected in the Western blot, spot 1 in Figure 3E represents most likely the unphosphorylated form of HP1β. This form had the weakest intensity of all HP1β isoforms detected and its intensity diminished even further after activation of BXB-ER in several experiments. Taken together these experiments suggest that whilst there is some increase in the phosphorylation of HP1α and HP1β proteins following the activation of BXB-ER, the association of HP1β with H3K9Me3 is not affected significantly (Fig. 3A). Thus the driving force behind HP1β re-localization, is not phosphorylation. Instead BXB-ER activation induced changes in repressive H3K9Me3 chromatin marks and subsequent association of HP1β to these new sites is sufficient to explain the observed HP1β redistribution (Fig. 2A).

**Figure 4:**
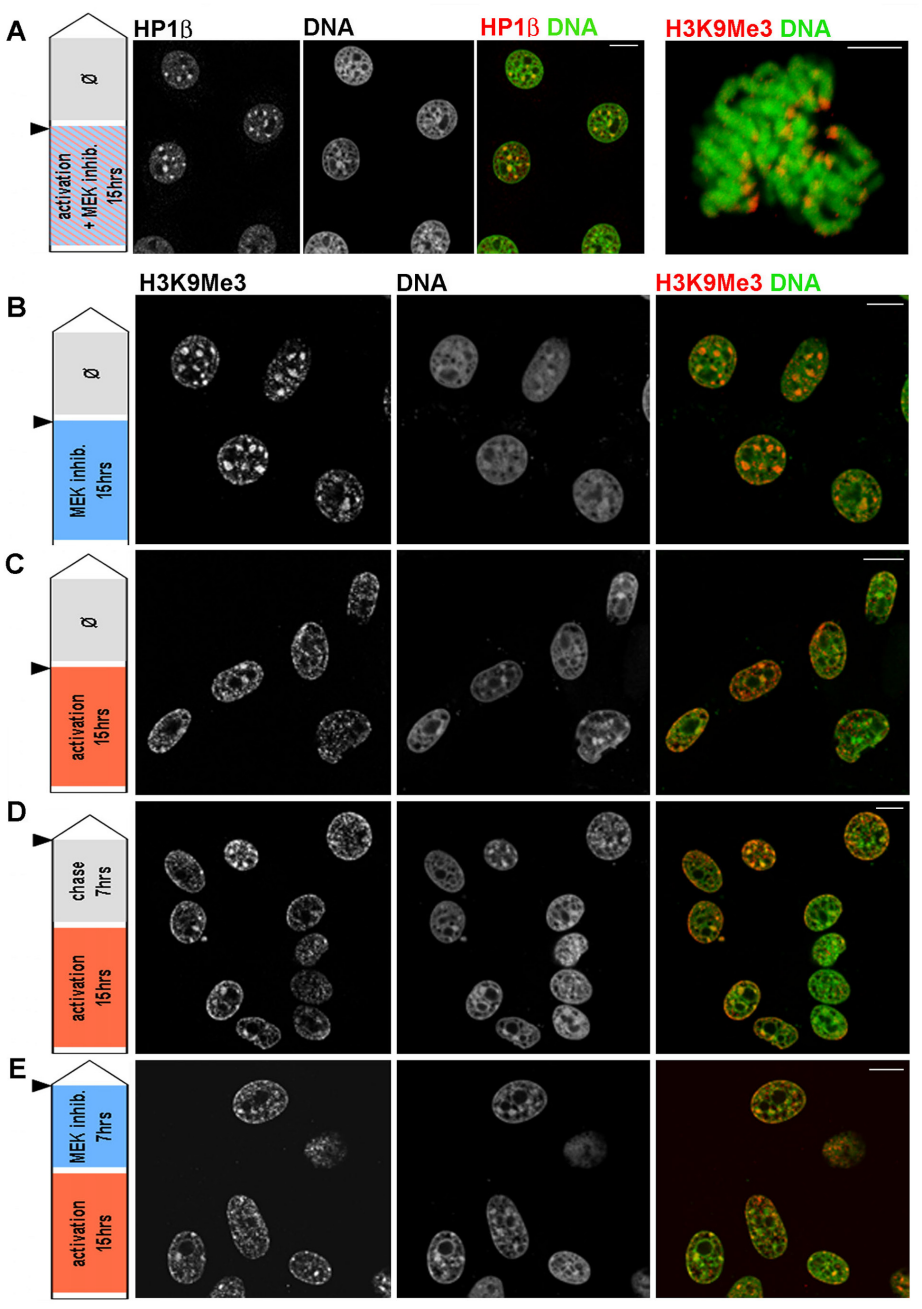
Epigenetic changes induced by BXB-ER activation are irreversible. 3T3BXB-ER cells were activated for 15 hours with estrogen, incubated with MEK inhibitor PD98059 or washed 3× and incubated with fresh medium or fresh medium with PD98059, as shown (left). Black arrowheads on the labeling schema indicate the time-point at which the cells were immunoprocessed (red) to reveal the distribution of HP1β (A) or H3K9Me3 (B–E). DNA was counterstained with Sytox (green). Analysis of single optical sections (A–E) shows that H3K9Me3 spreading is not reversible by chase with fresh medium (D) or PD98059 (E) for 7 hours. The last panel in (A) shows the H3K9Me3 signal of a typical metaphase spread from the same sample. Scale bars: 5 µm in (A), 10 µm in (B–E).

The reversibility of Raf/ERK induced chromatin remodeling was tested in time course experiments with the MEK inhibitor PD098059 [36]. Activation of BXB-ER in the presence of PD098059 eliminated the characteristic changes in H3K9Me3 and HP1β organization in interphase cells (compare Fig. 4A with Fig. 1B and Figs. 2A/D). The reversal of the effect of activated BXB-ER by PD098059 can also be seen in mitotic cells, where only pericentric H3K9Me3 staining on 3D reconstructed chromosomes can be observed, similar to the staining pattern observed in untreated 3T3 cells. Spreading of the H3K9Me3 stain to specific site throughout the genome and especially to the telomeres was not observed when cells with activated BXB-ER were also treated with PD098059 (compare Fig. 2D with Fig. 4A, panel on the right). Incubating 3T3BXB-ER with PD098059 in the absence of BXB-ER activation eliminated any residual BXB-ER and ERK activity (see Fig. 1A and [35]) and reverted the distribution of H3K9Me3 to 3T3 cell control status (Figs. 4B/2A). Following activation of BXB-ER for 15 hours, the redistribution of H3K9Me3 spread throughout nuclei (Fig. 4C). Once established, the structures formed by the H3K9Me3 redistribution remained unchanged even when cells were grown in either fresh medium without oestradiol (Fig. 4D) or with PD098059 (Fig. 4E). This is despite the fact that PD098059 can reverse the effect of BXB-ER activation on the activation of ERKs at any time (see Figure 1A, and Fig. 4A). The results for 12 and 24 hours chase periods with fresh medium or with PD098059 containing fresh medium (data not shown) were identical to the results of the 7 hours chase periods shown in Figs. 4D/E. This analyses show that once the epigenetic changes to H3K9Me3 are established they remain stable and unaffected by the BXB-ER activation status.

**Figure 5:**
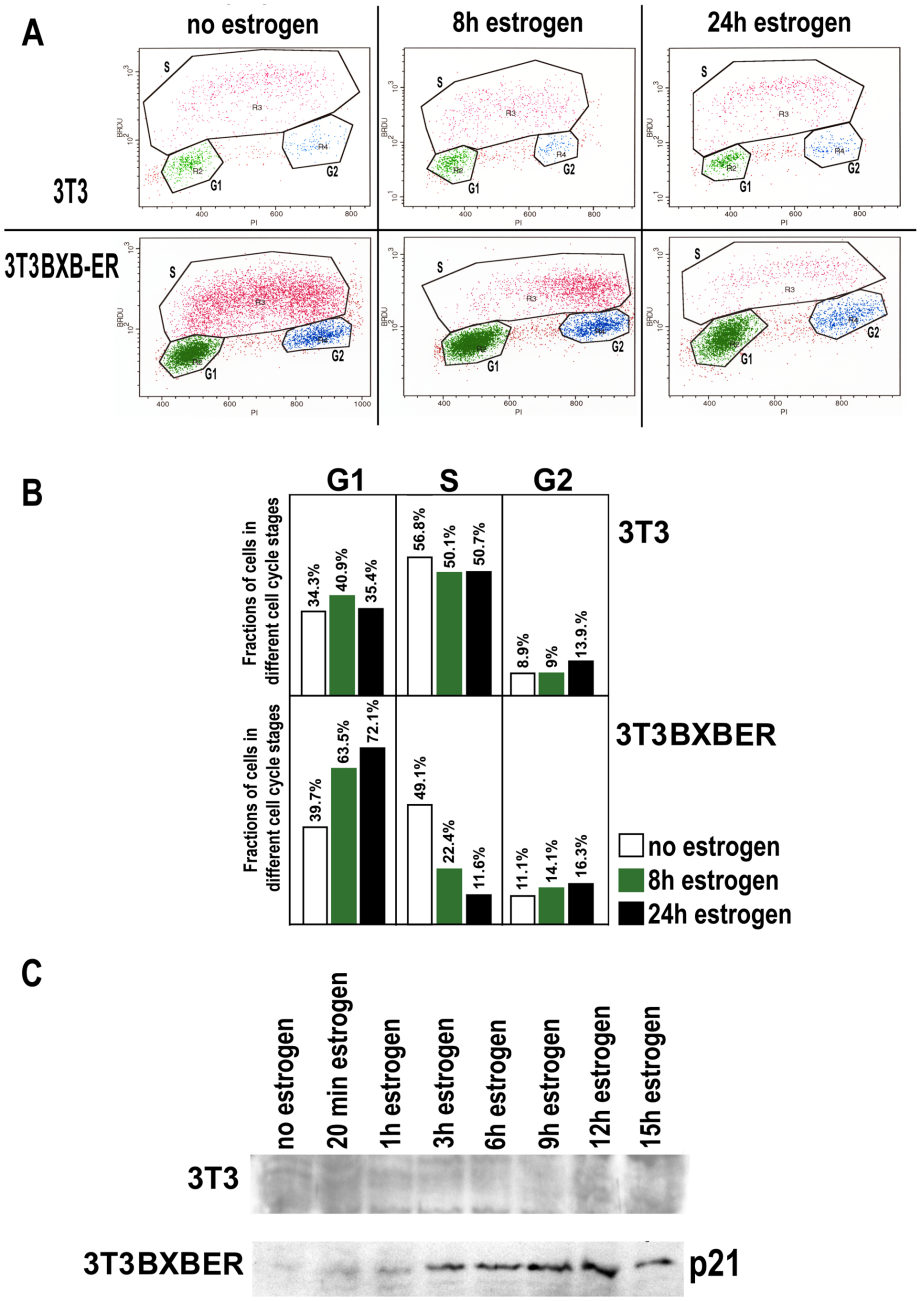
Cell cycle distribution of 3T3 and 3T3BXB-ER cells after prolonged activation of BXB-ER and BXB-ER induced accumulation of p21. (A) Cell cycle analysis of 3T3 and 3T3BXB-ER cells. Exponentially growing cells were left untreated (first panel) or activated with estrogen for 8 or 24 hours. Cells are gated into different cell cycle stages (as outlined in the figure) dependent on their total DNA content (Propidium Iodide intensity, F1 abscissa, ranging from 2N for cells in G1-phase to 4N for cells in G2-phase) and the amount of BrdU incorporated into the genomic DNA in the last 3 h before processing (ordinate, BRDU, low BRDU intensity for cells in G1- and G2-phase, high BRDU intensity for S-phase cells). (B) shows the numerical analysis of the FACS results - percentages are shown as percent of all gated cells. (C) Western blot for p21. Exponentially growing 3T3 and 3T3BXB-ER cells were left untreated or incubated for the indicated times with estrogen and blots were processed in parallel. 20 µg of protein from the various whole cell lysates were loaded into each lane. The parts of the blots where p21 migrates are shown.

Prolonged activation of Raf can have various results on cells, involving differentiation, proliferation and cell cycle arrest [3], [4], [8], [11]. We tested the effects of BXB-ER activation on the cell cycle in our cellular system by FACS (Fluorescence Activated Cell Scan) analysis of DNA incorporation and total DNA content (Fig. 5A). While estrogen treatment of 3T3 control cells for 8 hours has no effect on the distribution of cells in the cell cycle, the activation of BXB-ER by estrogen in 3T3BXB-ER cells induces a visible change within 8 hours of estrogen treatment. Fewer cells transit from the G1-phase into the S-phase of the cell cycle at this time point, resulting in a sparsity of cells in early S-Phase. After 24 hours of estrogen treatment the block in the cell cycle is even more pronounced and the amount of cells in the S-phase is lowered throughout the entire S-phase. Analysis of the numerical distribution of cells in the different stages of the cell cycle shows that estrogen has no effect on 3T3 cells (Fig. 5B). In contrast, the activation of BXB-ER and thus ERK1/2 in 3T3BX-BER cells by estrogen leads to a massive increase of cells in G1-phase of the cell cycle and to a massive reduction of cells in S-phase, while there is a modest increase of cells in the G2-phase (Fig. 5B) These effects on the cell cycle distribution are observed after 8 hours of estrogen treatment and become even stronger after 24 hours of estrogen treatment.

It has been reported previously that oncogenes can induce cell cycle arrest and senescence. In the case of Ras and Raf it has been shown that this cell cycle arrest can depend on the accumulation of p21 (Cip1/Waf1) (reviewed in [4], [11]). p21 is a key cell cycle regulator, which inhibits activation of cyclin-CDK2/4 complexes that are required for cells to pass from G1 to S phase of the cell cycle [44]. To test if BXB-ER activation induces the accumulation of p21 in our cellular system, we performed time course experiments of estrogen treatments followed by Western blots for p21 in 3T3BXB-ER cells and in control 3T3 cells (Fig. 5C). While p21 was undetectable in proliferating 3T3 cells with or without estrogen treatment as expected, the activation of BXB-ER in 3T3 BXB-ER cells resulted in the rapid accumulation of p21 (Fig. 5C). The progressive increase in p21 abundance in 3T3BXB-ER cells following estrogen treatment correlated with the accumulation of cells in the G1-phase (Fig. 5B) as would be expected for a p21 induced cell cycle arrest.

## Discussion

**Figure 6:**
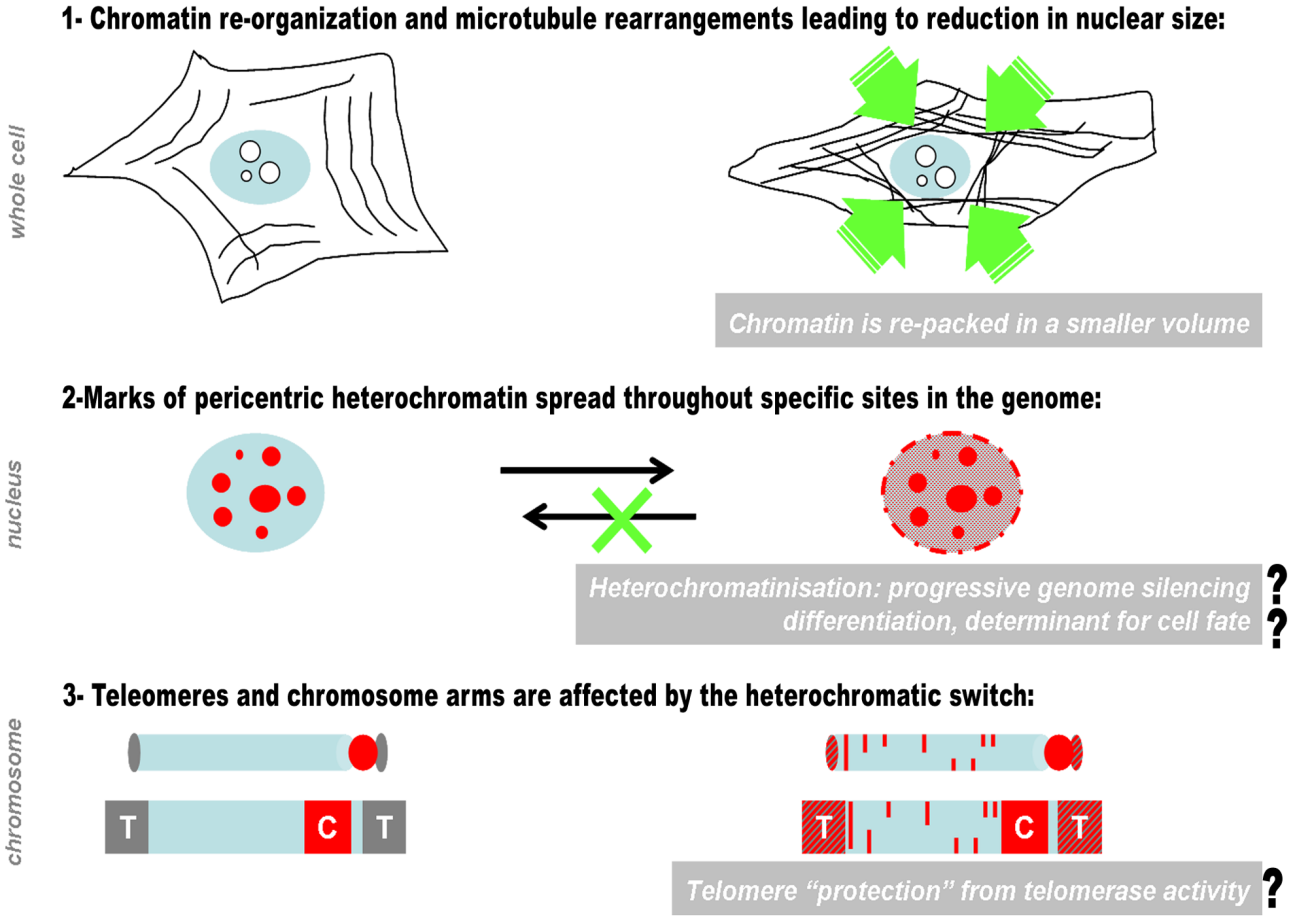
Proposed crosstalk between elevated Raf activity and genome heterochromatinization. 1- Prolonged activation of the Raf pathway (right) triggers nuclear rearrangements compared to cells with normal Raf activity (left). Following Raf activation, chromatin is condensed and folded into a smaller nuclear volume than in normal cells. At the same time, BXB-ER-induced microtubule bundling in the cytoplasm [35] might be involved in generating additional physical constraints, which correlate with reduced nuclear volume. 2- Irreversible spreading of heterochromatin marks (shown in red) might cause a progressive genome silencing, a mechanism that is linked to differentiation or growth inhibition. 3- Such BXB-ER induced epigenetic remodeling of chromatin occurs both, at sub-telomeric and at dispersed sites throughout the genome. We propose that this epigenetic switch is part of a mechanism that controls changes in transcription during cell cycle arrest.

This report links Raf/ERK activity to an epigenetic phenotype defined by the specific redistribution of the heterochromatic marker H3K9Me3 and its associated HP1 proteins. These changes in heterochromatin organization are important in the regulation of cell differentiation, senescence, aging and cellular transformation and might be instrumental for the function of Raf in these cellular processes (Fig. 6). Our results indicate that there might be several levels at which Raf activation influences heterochromatin components. Next to the stable induction of H3K9Me3, Raf activation induces some changes in the phosphorylation of the HP1 isoforms α and β (Figs. 3B/E). While we found a single phospho-isomer of HP1α, we detected 5 putative phospho-isoforms of HP1β. HP1α phosphorylation shows a transient increase after 20 min of BXB-ER activation while the unphosphorylated form of HP1β showed a slight decrease in abundance after prolonged BXB-ER activation, which implies a higher proportion of phosphorylation. Such an increase in phosphorylation is difficult to measure if the phosphorylations are spread over several phopsho-isoforms (Fig. 3E). This differential behavior of HP1α and HP1β might be a reflection of the slight differences in their primary sequence and their localization (reviewed in [26]). Further studies will have to show how Raf activation can change HP1 phosphorylation (neither HP1α nor HP1β carry any consensus sites for ERK phosphorylation) and what the functional consequences of these HP1 phosphorylations might be.

The BXB-ER induced epigenetic changes also result in modifications of chromosome structure and compromise the efficacy of metaphase chromosome formation (Fig. 2D/E). Enhanced formation of heterochromatin, like the one observed after induction of BXB-ER, is believed to compromise the efficacy of cell cycle progression, contributing to senescence [45]. Moreover, the observed changes also correlate with an altered cell cycle distribution, which is consistent with cycle arrest occurring within hours of BXB-ER activation (Fig. 5A/B). These observations are consistent with BXB-ER activation resulting in the induction of cellular senescence [46]–[49]. Alternative pathways of Raf induced senescence are known to involve p16^INK4A^/p14^ARF^ or p53 induced p21 (Cip1/Waf1) [47]. However, as the NIH 3T3 cells and their derivatives used in this study are known to have lost the *ink4* locus [50], [51], it is not surprising that p21 (Cip1/Waf1) accumulation is indeed induced after activation of BXB-ER (Fig. 5C), under conditions where a profound G1-phase arrest is observed (Fig. 5A/B).

H3K9Me containing senescence associated heterochromatin foci (SAHF) are a hallmark for oncogene induced senescence [45]. 30–40 SAHF are typically seen in the nuclei of senescent cells, where individual foci correspond to the condensed chromatin of single chromosomes. Interestingly, telomeric and centromeric chromatin domains make little contribution to the structure of SAHF. This applies to senescence induced by activated Ras, MEK or other oncogenes [52]–[54]. In contrast, the morphology of heterochromatin in interphase after BXB-ER activation is very different from the well-characterized SAHF phenotypes (e.g compare. Fig. 1B and [53]). Indeed the epigenetic phenotype we observe after BXB-ER activation is characterized by telomeres with strong H3K9Me3 staining (Fig. 2D/E). We thus conclude that prolonged activation of BXB-ER does not induce typical senescence, as defined by the formation of SAHF, but a different epigenetic phenotype.

Notably, the increased expression of p21 that is seen following prolonged BXB-ER activation is consistent with cell cycle arrest and the onset of senescence, even though the epigenetic phenotype we observe is not typical of that seen during the classical forms of oncogene induced senescence. In future studies it will be interesting to establish if both states are induced using the p53/pRB pathway as previously characterized [49], [54] and how p21 induction and the epigenetic changes induced by BXB-ER are connected. Interestingly, in fibroblasts, p21 accumulation can be induced by post-translational mechanisms [55] as well as by dysfunctional telomeres [56]. As BXB-ER induces strong H3K9Me3 at telomeres and this might interfere with telomeric function, our results are compatible with several alternative mechanisms by which p21 levels could be regulated [44].

The acquisition of robust pericentric-like markers at telomeres after prolonged BXB-ER activation might be expected to interfere negatively with telomere maintenance [57]–[59] and enhance telomere shortening [60]. This in turn points towards a potential role for prolonged Raf activation on cell proliferation capacity in tumorigenesis [61], [62], a process in which Raf is known to be involved [63]–[66]. Moreover, inhibition of telomerase function could counteract the effect of cell transformation and explain the negative effects of activated Raf, which are seen in a variety of cancer cells [67], [68]. This possibility is supported by the properties of tumours with mutationally activated B-Raf [48] and is unlikely to reflect abnormally high levels of Raf activity [69]. Results described here are consistent with alterations in telomere function, providing a potential mechanism by which prolonged Raf activation is involved in regulation of cell growth and transformation.

The telomere position effect (TPE) is an important mechanism for silencing genes adjacent to telomeric regions. As prolonged BXB-ER activation induces the accumulation of H3K9Me3 in telomeric regions as well as in other distinct regions throughout the genome (Fig. 2D/E) and since H3K9Me3 is an epigenetic marker involved in silencing transcription [19], [26], [31], [70] our experiments point to the possibility that Raf-induced epigenetic changes might contribute to gene silencing via TPE. While telomere dysfunction is linked to genomic instability and cancer progression [57], [71], enhanced H3K9Me3 and telomere shortening are essential during differentiation [72]–[74]. Prolonged Raf activation is involved in differentiation, for which nuclear targets are essential and ERK translocation to the nucleus is a necessity for differentiation [10], [12]. However, our results are consistent with the possibility that changes in the structure of sub-telomeric chromatin lead to alterations in telomere function, which contribute to Raf/ERK induced differentiation (Fig. 6).

At the moment we can only speculate about the possible biological consequences of the BXB-ER induced changes in heterochromatin and alterations of chromosome structure described in this report (Fig. 6). However, based on our observations, we suggest that future studies of Raf/ERK function should evaluate how global changes in the organization of post-translational chromatin modifications link the signaling pathway to mechanisms that control down-stream patterns of gene expression.

## Materials and Methods

### Cell culture, constructs, fresh weight determination and ^32^P labeling

NIH 3T3 cells and 3T3BXB-ER cells were grown at 37°C in an incubator with 5% CO_2_ in DMEM (high Glucose) with 10% FCS. 3T3BXB-ER cells are a mixture of 5 clones derived from NIH 3T3 cells after stable transfection with a BXB-ER producing construct [35]. BXB-ER is a fusion protein containing the kinase domain of C-Raf, which is only active in the presence of estrogen and is described in reference [35]. All activations of BXB-ER were performed at a final concentration of 5 µM estrogen. All control cells were treated with 0.1% ethanol (carrier). Where indicated, cells were treated with 50 µM PD98059 or carrier alone (0.1% Me_2_SO) or 20 µM UO-126 or carrier alone (0.2% Me_2_SO). For the determination of the fresh weight of cells, 1×10^7^ cells for each condition were scraped from cell culture dishes and their weight determined after pelleting at 1 500× g for 10 min. 4 independent experiments were performed. For in vivo labeling with ^32^P, cells were washed 3× with phosphate free medium and labeled with carrier free ^32^P-orthophsophate exactly as described before [35].

### Immunofluorescence, confocal microscopy and FACS analysis

Cells were grown on coverslips and fixed in PBS-PFA (Phosphate Buffered Saline-Paraformaldehyde) (5 min at 20°C), permeabilized in PBS-Triton X-100 (0.5%) for 15 min at 20°C before blocking in PBS-BSA (2%) for 60 min. Primary antibodies against HP1β (Euromedex, 1∶400) and fibrillarin (Abcam, 1∶400) were diluted in PBS-BSA (Bovine Serum albumin) (2%) and incubated at 4°C overnight. After 3 washes in PBS-Tween (0.02%), the coverslips were incubated with secondary antibodies coupled to Cy3 (Jackson ImmunoResearch 1∶300,) or Alexa Fluor 488 (Molecular Probes 1∶500) for 1 hour at 20°C in the dark. After 3 washes in PBS-Tween (0.02%) the samples were briefly post-fixed in PBS-PFA 1%. Counterstaining of DNA was performed with 0.25 µM Sytox green (Invitrogen) and samples were finally mounted with Vectashield (Vector Laboratories). A confocal microscope (Zeiss LSM510, with a plan apochromat 63× objective, DIC 1.4NA) was use to capture single optical sections. For 3D views, slides with tape spacers, to avoid cell/nucleus squashing, were used to mount the coverslips. The basis for the reconstructions were 300 nm z-stepped stacks, which were combined using Zeiss and Imaris software. For phenotype quantification and the measurement of nuclear diameters random field single optical slice pictures were taken and cells were put into bins according to criteria outlined in the text. Specifically, the staining patterns for H3K9Me3 and HP1β were defined as follows. The bin “chromocentres only” was defined by the absence of none-particulate and homogeneous immunofluorescence signal in the nucleus; i.e. less than 40% of the area of the optical slice of the nucleus showed a visible signal and more than 80% of this signal was due to strong granular stains. The bin “chromocentres and nucleoplasm” was defined by a signal in more than 40% of the area of the nucleus and more than 20% of the area with a signal was due to homogeneously stained nucleoplasm as opposed to strong granular stains.

Cell cycle analysis of 3T3BXB-ER cells was performed according to [75]. In short, BrdU was added to the medium 4 hours before processing for FACS analysis. Cells were fixed, membranes permeabilized and samples were then treated with propidium iodide, before being analyzed on a FACS Calibur (Becton Dickinson).

### 2D SDS PAGE Western Blotting and Immunoprecipitation

2D gel electrophoresis was performed as described before [35] with some modifications. 3×10^6^ cells in 10 cm culture dishes were washed twice in ice cold PBS, scraped from the plate with an eraser and washed once more in 0.5× PBS before being lysed in 300 µl sample buffer (11 M urea, 4% CHAPS, 40 mM Tris, 1% dithioerythritol, 2.5 mM EDTA, 2.5 mM EGTA). DNA was removed by centrifugation at 100 000× g (60 min) and the samples (representing 70–200 µg from 1–3×10^6^ cells) were loaded (after adding loading ampholytes pH 3–10, 0.5%, GE Healthcare) on custom made narrow pH range IPG strips on plastic supports (see figure legend). After running for 120 kVH in a stepwise manner the gels were processed as described in [35]. The 2D SDS PAGE gels were silver stained and dried for exposure to a PhoshorImager, Typhoon. ImageQuant software was used for quantification of ^32^P signals. For Western blots of 2D gels an 8×10 cm piece of the 2D gel with the region of interest in the middle was cut out and processed as described before [38] using primary antibodies against HP1β (Euromedex, 1∶500). Western blots from SDS PAGE gels were performed as described in [38], using antibodies against ERK1/2 phosphorylated on residues Thr 202/Tyr 204 (Cell Signaling Technology, 1∶1000), ERK (Sigma, 1∶10 000), HP1β (Euromedex, 1∶500) and p21 (Abcam, 1∶100). To ensure equal protein load from different samples, protein contents of whole cell lysates were measured by the method of Bradford and equal protein amounts were loaded. Blots were re-probed after stripping for 30 min at 50°C in 2% SDS, 50 mM Tris pH 8.8 and 1% β-Mercaptoethanol. Immunoprecipitations were performed exactly as described before [38] using primary antibodies against H3K9Me3 (Abcam, 1∶50). Immunoprecipitates were separated and subjected to Western blots in parallel to cellular lysates.

### Mass spectrometry

Gel signals from scanned silver stains and ^32^P signals were aligned using ^14^C radioactive ink in the corners of the gel. The Adobe Photoshop two layers/fade function was used to reveal phosphoproteins corresponding regions on the silver stain gel image and spots of interest were labeled for subsequent extraction. The centre of the spots were cut from the gels and destained [76]. Proteins in the gel pieces were digested with trypsin and analyzed by MALDI-ToF MS (Matrix Assisted LASER Desorption/Ionization Time of Flight Mass Spectrometry) as described [76], using a Bruker Reflex III instrument.
